# Sotorasib resistance in *KRAS* G12C-mutant invasive mucinous adenocarcinoma with implications for VEGF-A

**DOI:** 10.1038/s41698-025-00953-2

**Published:** 2025-05-27

**Authors:** Hiraku Yanada, Ryohei Yoshida, Ryotaro Kida, Kiichi Nitanai, Maya Ikeda, Kazunori Nagasue, Taeka Naraoka, Masashi Ueda, Takashi Watanabe, Ryota Shigaki, Yasuhiro Umekage, Yoshinori Minami, Toshihiro Nagato, Manami Hayashi, Sayaka Yuzawa, Mishie Tanino, Takaaki Sasaki

**Affiliations:** 1 Department of Internal Medicine, Division of Respiratory Medicine and Neurology, Asahikawa Medical University, Asahikawa, Hokkaido 078-8510 Japan; 2 Clinical Research Center, Keiyukai Yoshida Hospital, Asahikawa, Hokkaido 070-0054 Japan; 3 Department of Pathology, Asahikawa Medical University, Asahikawa, Hokkaido 078-8510 Japan; 4 Department of Diagnostic Pathology, Asahikawa Medical University Hospital, Asahikawa, Hokkaido 078-8510 Japan

**Keywords:** Non-small-cell lung cancer, Cancer, Lung cancer

## Abstract

Invasive mucinous adenocarcinoma (IMA) is a rare subtype of lung adenocarcinoma with a poor prognosis. Compared to non-small cell lung cancer, IMA more frequently harbors *KRAS* mutations in the order p.G12V, p.G12D, and p.G12C. This report describes a patient with a *KRAS* p.G12C-mutant IMA treated with sotorasib. To date, no studies have investigated the therapeutic efficacy or resistance mechanisms of sotorasib in IMA. The patient was treated with carboplatin and pemetrexed, followed by sotorasib upon disease progression. While the primary lung lesions responded well, metastatic thoracic lymph node lesions continued to increase. A pathological autopsy was performed with the family’s consent to investigate potential resistance mechanisms. RNA sequencing and additional analyses revealed increased VEGF-A expression in metastatic lymph node lesions, suggesting a role in sotorasib resistance. These findings provide insights into the potential molecular mechanisms underlying treatment resistance in *KRAS* p.G12C-mutant IMA.

## Introduction

Invasive mucinous adenocarcinoma (IMA) is a rare subtype of lung adenocarcinoma, accounting for approximately 3–10% of all cases^1,2^. IMAs are associated with a poor prognosis and characterized by tumor cells with a high columnar shape and abundant intracytoplasmic mucus. One contributing factor to their unfavorable outcome is low PD-L1 expression, which limits the efficacy of immune checkpoint inhibitors^3,4^. Approximately 70% of IMAs harbor activating mutations in the Kirsten rat sarcoma viral oncogene homolog (*KRAS*) gene, with *KRAS* p.G12C mutations being less common than *KRAS* p.G12V or p.G12D mutations^5^. Thoracic lymph node metastases are less common in IMAs compared with other subtypes of lung adenocarcinoma^6^. Sotorasib, a first-in-class *KRAS* p.G12C inhibitor, has shown significant efficacy in treating *KRAS* p.G12C-mutant non-small cell lung cancer (NSCLC)^7^. However, little is known about its therapeutic potential or resistance mechanisms in IMA, as no prior reports have explored these aspects in this rare histological subtype.

Here, we present a unique case of a *KRAS* p.G12C-mutant IMA treated with sotorasib. Although the initial response was promising, the disease eventually progressed with thoracic lymph node metastasis. Postmortem analysis revealed upregulation of vascular endothelial growth factor A (VEGF-A), suggesting a potential mechanism of resistance to sotorasib and highlighting the therapeutic challenges in managing this understudied disease.

## Results

### Clinical data of patients with IMA at our hospital

A retrospective review of cases diagnosed with IMA based on surgical or biopsy specimens at our hospital between January 2015 and March 2023 revealed that only 2 of 32 patients exhibited mediastinal lymph node metastases at the time of initial diagnosis (Table S1). PD-L1 testing was not performed in 24 cases. Among the tested cases (*n* = 8), none showed high PD-L1 expression, and all demonstrated either low or negative expression.

Multiplex companion diagnostic (CDx) testing with the Amoy DX or Oncomine DX Target Test was conducted for 9 of the 32 patients, and *KRAS* gene mutation testing using PCR-rSSO was conducted for 7 of the 32 patients. Among the 16 cases tested for genetic mutations, 12 were positive for *KRAS* mutations, with mutation subtypes such as p.G12C, p.G12V, and p.G12D being frequently observed (Fig. S1). These findings are consistent with those of previous reports^4–6^.

### Clinical history

**Fig. 1:**
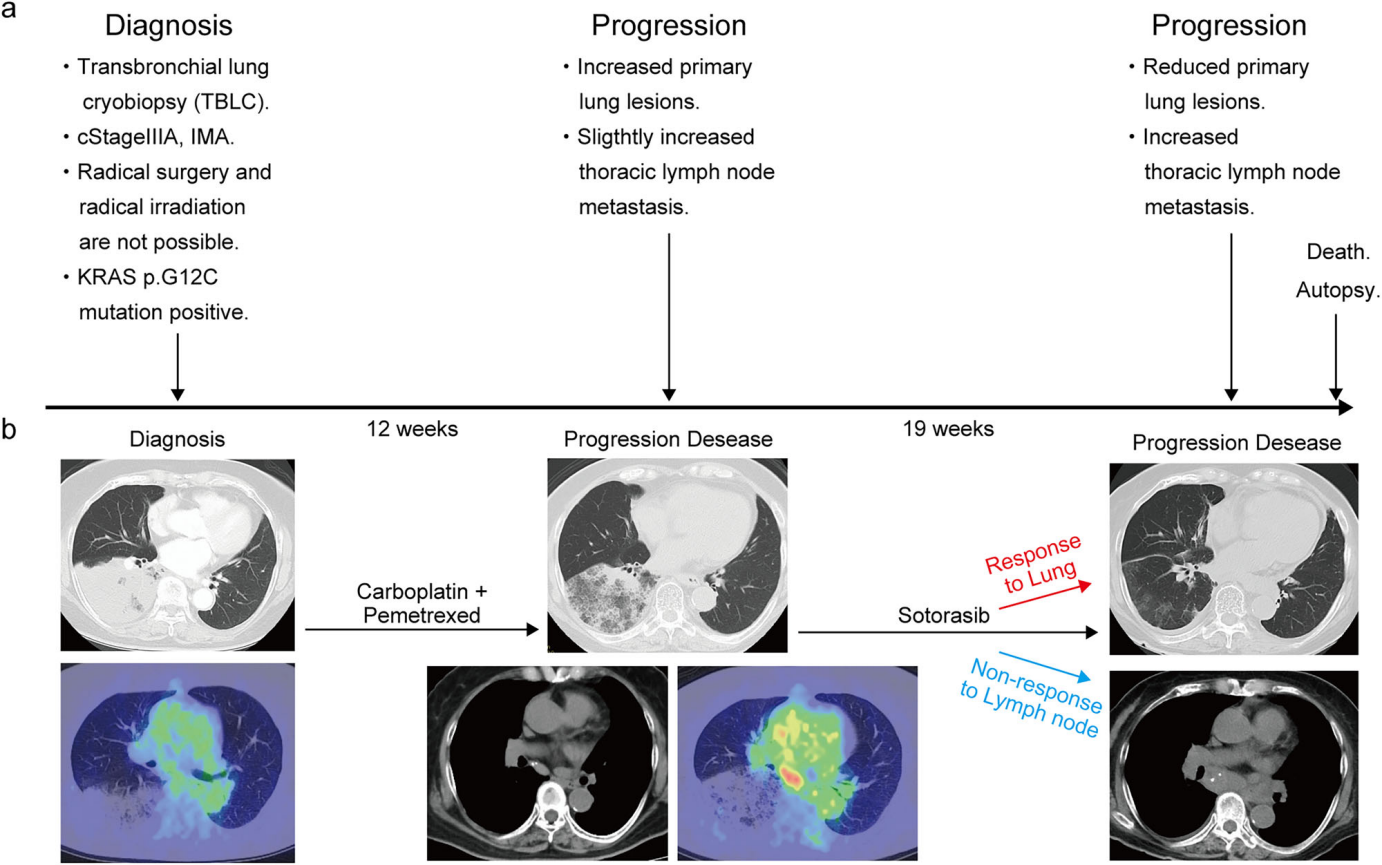
Patient’s clinical course. **a** Overview of the patient’s diagnosis and treatment timeline. **b** Radiographic changes and corresponding treatment details.

A 78-year-old woman with no history of smoking was referred to our hospital with pneumonia that was unresponsive to antimicrobial therapy. Chest CT revealed extensive infiltrative shadows in the lower lobe of the right lung. A transbronchial lung cryobiopsy was performed, leading to a diagnosis of IMA with a *KRAS* p.G12C mutation confirmed by CDx testing using Oncomine DX Target Test. The clinical stage was cT4N0M0, corresponding to cStage IIIA (Fig. 1a). Initially, three cycles of carboplatin plus pemetrexed were administered; however, the primary tumor enlarged and metastasized to the mediastinal lymph nodes, indicating disease progression (Fig. 1b). Subsequently, sotorasib (960 mg/day) was initiated as second-line therapy. After 103 days of treatment, the sotorasib dose was reduced due to the development of grade 2 nausea. By day 130, the primary lesion in the right lower lobe had decreased in size; however, disease progression, marked by mediastinal lymph node enlargement, necessitated treatment discontinuation (Fig. 1b). The patient was later admitted to our hospital with pneumonia and severe dehydration. Despite treatment, her general condition deteriorated, and she passed away. A pathological autopsy was performed with the consent of her family.

### Analysis of autopsy specimens

Histopathologically, both lung and thoracic lymph node lesions showed atypical cells with irregularly shaped enlarged nuclei with enriched chromatin and abundant intracytoplasmic mucus. These cells demonstrated papillary proliferation and alveolar epithelial replacement, floating in the mucus lake. Immunohistochemistry (IHC) staining results were as follows: CK7 (+), CK20 (partially +), CDX2 (−), TTF-1 (−), Napsin A (Lung: partially +, Lymph node: −), and MUC5AC (+), findings strongly suggestive of IMA.

**Fig. 2:**
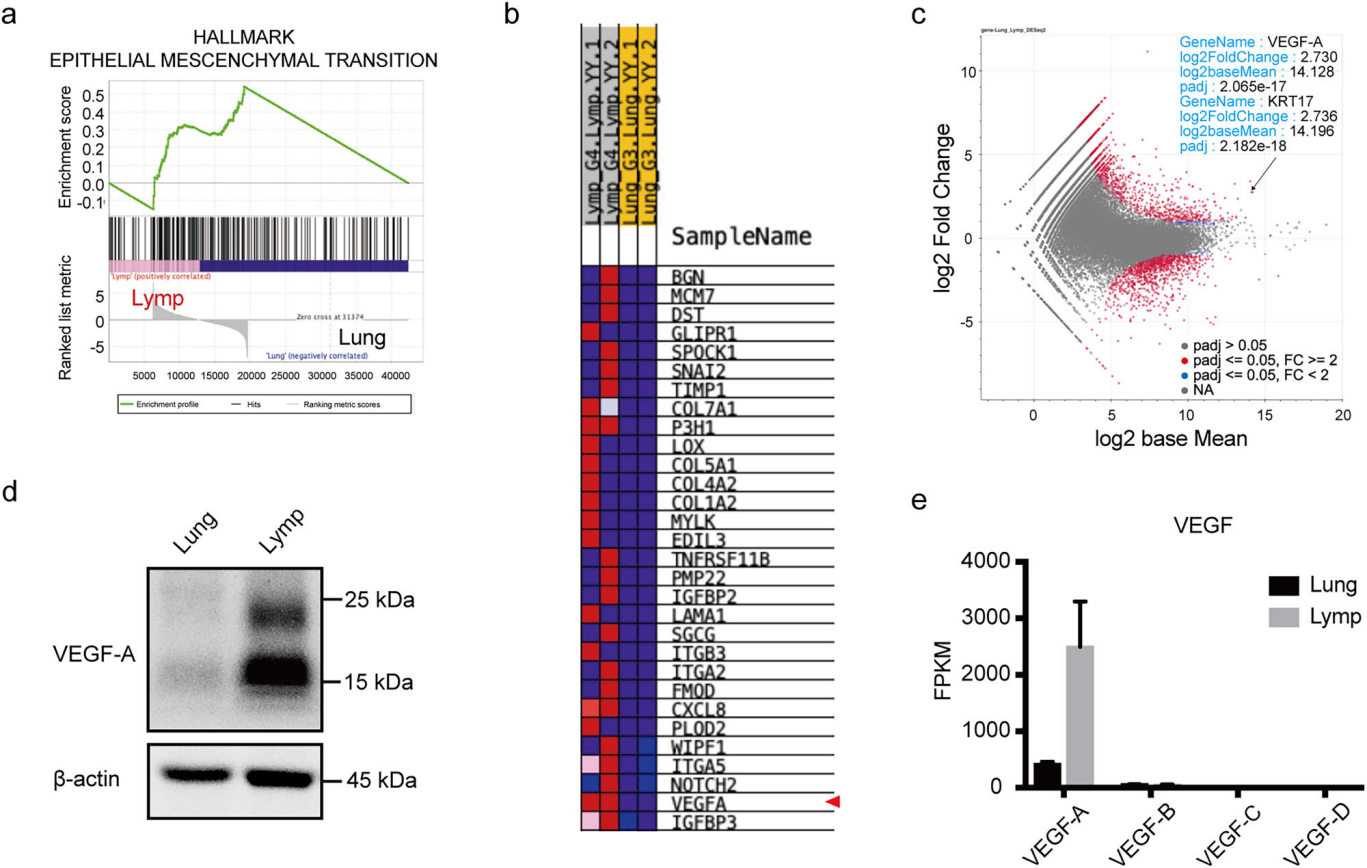
Analysis of autopsy specimens. **a** GSEA was performed using RNA sequencing data from autopsy specimens of primary lung cancer lesions and metastatic thoracic lymph node lesions. In thoracic lymph node lesions, the Hallmark gene set database indicates positive regulation of EMT signaling categories. **b** Heat map displaying the 30 core-enriched genes. **c** MA plot illustrating molecules associated with EMT, showing elevated VEGF-A levels. Data are expressed as the log ratio of intensity relative to the mean intensity across both samples. **d** Comparison of gene expression levels for VEGF-A, B, C, D in FPKM. **e** Western blot analysis of lysates extracted from primary lung and lymph node lesions in autopsy specimens. GSEA gene set enrichment analysis, RNA ribonucleic acid, EMT epithelial-mesenchymal transition, VEGF-A vascular endothelial growth factor A, MA plot mean-average plot, FPKM fragments per kilobase of exon per million mapped reads.

**Fig. 3:**
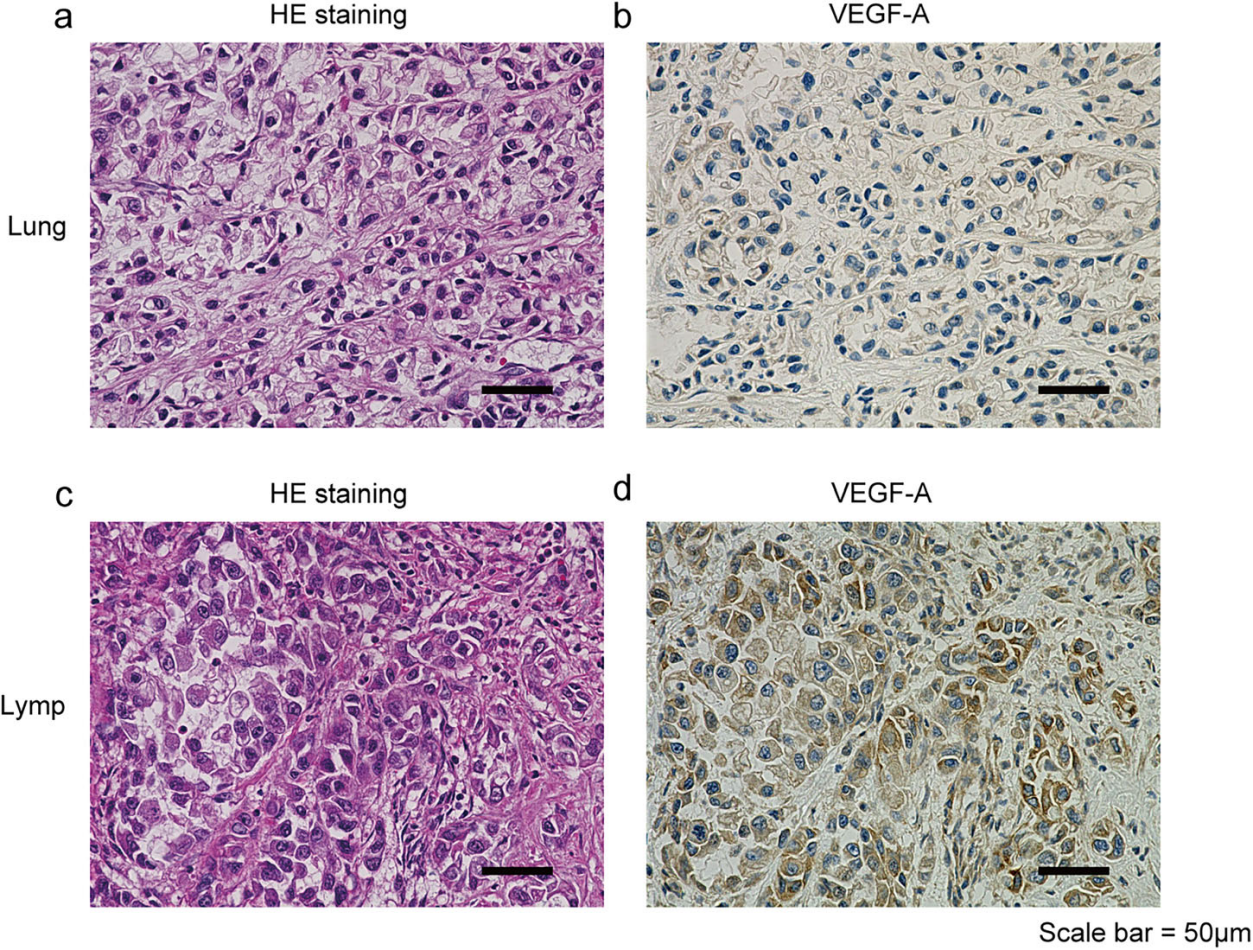
Immunohistochemical analysis of autopsy specimens. **a** HE staining of primary lung lesions. **b** VEGF-A staining of primary lung lesions. **c** HE staining of thoracic lymph node metastases. **d** VEGF-A staining of thoracic lymph node metastasis. HE hematoxylin and eosin, VEGF-A vascular endothelial growth factor A.

In this case, although the primary lung lesion sizes remained reduced following sotorasib treatment, the number of mediastinal lymph node lesions increased. Therefore, we compared and analyzed these findings to investigate the mechanisms underlying drug resistance. Next-generation sequencing analysis of the extracted DNA confirmed the presence of the *KRAS* p.G12C mutation in both the lung and lymph node lesions, with no mutations associated with sotorasib resistance identified (Table S2). RNA sequencing was performed on extracted RNA, followed by gene set enrichment analysis (GSEA). This analysis revealed a significant upregulation of epithelial-mesenchymal transition (EMT) signaling in lymph node lesions compared to lung lesions (Fig. 2a, Fig. S2a). However, protein analysis of the extracted samples showed that both E-cadherin and vimentin were expressed in lung and lymph node lesions, with no significant differences related to EMT (Fig. S2b). These findings were further supported by IHC, which showed no differences in E-cadherin and vimentin staining between the primary lung lesion and mediastinal lymph node metastases (Fig. S3a–f). To further investigate the mechanism of sotorasib resistance, we examined the EMT signature components identified by GSEA. VEGF-A expression was also observed (Fig. 2b, c). Differential expression analysis using Fragments Per Kilobase of exon per Million mapped reads (FPKM) revealed that VEGF-B, VEGF-C, and VEGF-D were not upregulated in either lung or lymph node lesions, whereas VEGF-A expression was specifically increased (Fig. 2d). This upregulation of VEGF-A in lymph node lesions was further confirmed by protein analysis (Fig. 2e). Immunostaining for VEGF-A revealed distinct differences between thoracic lymph node metastases and primary lung lesions (Fig. 3a–d). In particular, staining was more pronounced in cancer cells than in normal cells or the stroma. In addition, CD31 staining was performed, and the number of stained vessels per 10 high-power field in the center of each tumor focus was counted; 7 for primary lung lesions and 18 for thoracic lymph node lesions (Fig. S3g–j). These findings suggest that thoracic lymph node lesions are more angiogenically active, likely due to elevated VEGF-A expression.

## Discussion

This case report provides insights into the therapeutic efficacy of sotorasib in *KRAS* p.G12C mutation-positive IMA and highlights a potential resistance mechanism. Although the clinical benefit of sotorasib in *KRAS* p.G12C mutation-positive NSCLC has been established, its efficacy and resistance patterns in IMA remain largely unexplored.

Hay et al. first described the role of EMT in cancer metastasis in the 1980s^8^. Since then, extensive research has linked EMT to various oncogenic processes, including acquired resistance to EGFR-TKIs in lung cancer^9^. More recently, EMT has been implicated as a resistance mechanism following sotorasib treatment in *KRAS* p.G12C-positive NSCLCs^10^. However, no studies have specifically examined the resistance mechanisms or therapeutic efficacy of sotorasib in *KRAS* p.G12C mutation-positive IMA. In this case, sotorasib was administered following disease progression on carboplatin plus pemetrexed. Although effective against lung lesions, sotorasib exhibits limited efficacy against mediastinal lymph node metastases. RNA sequencing revealed significantly elevated EMT-related signaling in lymph node metastases, with a marked increase in VEGF-A expression, with GSEA confirming these findings. Western blotting showed elevated E-cadherin and vimentin levels in both the lung and lymph node lesions, suggesting partial EMT. IHC yielded similar results, with no clear distinction between lung and lymph node lesions in terms of EMT expression.

EMT is a complex process influenced by diverse gene expression programs across different cancer types and microenvironments^11^. Our findings suggest that although EMT-related signals are elevated in lymph nodes, protein-level analyses alone may not fully capture their contribution to resistance. VEGF-A, which is significantly upregulated in lymph node metastases, has emerged as a potential driver of sotorasib resistance in *KRAS* p.G12C mutation-positive IMA.

Previous studies have demonstrated that VEGF-A expression in EMT-driven tumors enhances angiogenesis and metastasis, supporting its potential role in resistance^12^. Clinically, bevacizumab, an anti-vascular endothelial growth factor (VEGF)-A antibody, has shown efficacy in IMA^13^, although its precise mechanism of action remains unclear. It is plausible that VEGF-A overexpression represents a resistance mechanism, warranting further exploration of VEGF-A-targeted therapies in combination with sotorasib.

DNA sequencing revealed a unique *SYNE1* p.E4060D mutation in the lymph node metastases. Resistance to sotorasib has been linked to multiple mechanisms, including *MET* amplification; activating mutations in *NRAS*, *BRAF*, *MAP2K1*, and *RET*; oncogenic fusions involving *ALK*, *RET*, *BRAF*, *RAF1*, and *FGFR3*; and loss-of-function mutations in *NF1* and *PTEN*^14^. However, the relevance of *SYNE1* mutations in this case remains uncertain.

In *EGFR*-mutated NSCLCs, VEGF upregulation has been implicated in resistance to EGFR-TKIs, and anti-VEGF-A antibodies have demonstrated clinical benefit^15^. Similarly, EMT and VEGF-A-driven resistance mechanisms have been observed with osimertinib, where combination therapy with anlotinib suppressed VEGF-A and reversed EMT-associated resistance^16^. These findings suggest that a combinatorial approach targeting VEGF-A alongside sotorasib may have yielded better outcomes for lymph node metastases in this case.

This study has some limitations that should be considered when interpreting the findings. First, as this is a single-case report, broader conclusions require validation in larger cohorts. Second, although RNA sequencing provided valuable insights, the findings have not been extensively validated at the functional level. Protein expression analysis using Western blotting and IHC did not reveal clear distinctions in EMT-related markers between lung and lymph node lesions, suggesting that additional resistance mechanisms may be involved. Additionally, while VEGF-A has emerged as a potential contributor to resistance, clinical evidence supporting anti-VEGF-A therapy in IMA remains limited. Prospective trials are needed to determine its efficacy in this setting.

This case suggests that sotorasib is effective against *KRAS* p.G12C-positive IMA but may exhibit limited efficacy in lymph node metastases, potentially due to VEGF-A-mediated resistance. EMT-related signaling and VEGF-A overexpression may play key roles in this resistance. Further studies are warranted to validate these findings and explore therapeutic strategies that incorporate VEGF-A inhibition to enhance the efficacy of sotorasib in IMA.

## Methods

### Pathological autopsy

Pathological autopsies were performed by the Pathology Department of Asahikawa Medical University Hospital. Following the cessation of vital functions, the postmortem interval at room temperature was 11 h and 53 min. Before conducting the autopsies, COVID-19 PCR testing was performed, and the results were confirmed to be negative.

Tissue specimens were fixed in 10% neutral buffered formalin for approximately 24 h, processed using a tissue processor, and dehydrated in 100% ethanol. The sections were cleared with xylene, embedded in paraffin, and stored at room temperature. Tissue samples were preserved as formalin-fixed, paraffin-embedded (FFPE) blocks to preserve tissue morphology over time and facilitate histological analysis. RNA and protein were extracted from the tissue samples for further analysis.

### DNA analysis

Targeted amplicon sequencing was performed using the Ion AmpliSeq Comprehensive Cancer Panel (Thermo Fisher Scientific, Cat.#. 4477685), a previously validated panel for genetic analysis. Briefly, 10 ng of genomic DNA (gDNA) was amplified by PCR using the Ion AmpliSeq Library kit 2.0 (Thermo Fisher Scientific, Cat.#. 4480441). Sequencing was performed on an Ion PGM System following the manufacturer’s protocol.

### RNA-seq analysis

RNA sequencing, including library preparation, sequencing, mapping, gene expression analysis, and gene ontology (GO) enrichment analysis, was conducted using DNAFORM software (Yokohama, Kanagawa, Japan).

The quality of total RNA was assessed using a Bioanalyzer (Agilent) to determine the RNA Integrity Number (RIN). Poly(A) + RNA enrichment was performed using the NEBNext Poly(A) mRNA Magnetic Isolation Module (New England BioLabs). Double-stranded cDNA libraries (RNA-seq libraries) were prepared using the SMART-Seq Stranded Kit (Clontech) and the MGIEasy Universal Library Conversion Kit (App-A) (MGI Tech), following the manufacturer’s instructions.

RNA-seq libraries were sequenced using paired-end reads (150 nt of read 1 and read 2) on a DNBSEQ-G400RS instrument (MGI Technology). The raw reads were trimmed and quality-filtered using Trim Galore! (version 0.6.7), Trimmomatic (version 0.39) and Cutadapt (version 3.7). Trimmed reads were then mapped to the human GRCh38 genome using STAR software (version 2.7.10a). Reads mapped to annotated genes were counted using featureCounts (version 2.0.1).

FPKM values were calculated by normalizing mapped reads to total counts and transcripts. Differentially expressed genes were identified using the DESeq2 package (version 1.20.0). Genes detected by DESeq2 with a baseMean > 5 and fold-change < 0.25, or baseMean > 5 and fold-change > 4 were selected for GO enrichment analysis using the clusterProfiler package (Yu et al., OMICS 2012, 16:5).

### Western blot

Cells were lysed in RIPA lysis and extraction buffer (Thermo Fisher Scientific, Cat.#. 89900) containing 1× protease inhibitors (Roche, Cat.#. 11-836-145-001) and phosphatase inhibitors (50 μmol/L NaF and 100 μmol/L Na_3_VO_4_).

The primary antibodies used were E-cadherin (Cell Signaling Technology, Cat.#. 3195), vimentin (Cell Signaling Technology, Cat.#. 5741), VEGF-A (Cell Signaling Technology, Cat.#. 50661), and β-actin (Cell Signaling Technology, Cat.#. 4967). The secondary antibodies were anti-rabbit IgG and horseradish peroxidase (HRP)-conjugated antibodies (Cell Signaling Technology, Cat.#. 7074).

Anti-rabbit IgG and HRP-linked antibodies were diluted at 1:2500, while all other Cell Signaling Technology antibodies were diluted at 1:1000. Blot imaging was performed using a LAS-500 system (Fujifilm).

### Immunohistochemical staining

Formalin-fixed paraffin-embedded (FFPE) specimens were prepared from the autopsy samples and sectioned into 4-μm-thick slices.

Anti-E-cadherin antibody (Dako, Catalog no. NCG-38, 1:100) and anti-vimentin antibody (Dako, Catalog no. Vim3B4, 1:500) were used as primary antibodies. FFPE samples were stained using the BOND-III system (Leica) with the BOND Polymer Refine Detection (Leica) as the antigen retrieval solution. Representative images were acquired using an ECLIPSE Ni microscope (Nikon, Tokyo, Japan).

Anti-VEGF-A antibody (Abcam, Catalog no. ab1316, 1:200) was also used as a primary antibody. For this antibody, FFPE samples were stained using the VENTANA Discovery ULTRA system (Roche Diagnostics) using Cell Conditioning 1 buffer ULTRA (Roche Diagnostics) as the antigen retrieval solution and the VENTANA Ultra View Universal DAB Detection Kit (Roche Diagnostics). Representative images were acquired using a BZ-X710 microscope (Keyence, Osaka, Japan).

### Study approval

The patient’s family provided written consent for the pathological autopsy, case report publication, and genetic analysis. This study was conducted in accordance with the principles of the Declaration of Helsinki. Genetic and other analyses were performed with the approval of the Institutional Review Board (IRB) of Asahikawa Medical University on March 11, 2025 (approval number: 24169).

## Supplementary information

Supplementary_Information

## Data Availability

The datasets generated and/or analyzed in this study are available from the corresponding author upon reasonable request. The raw and processed data have been deposited in the Gene Expression Omnibus (GEO) under accession number GSE295712.

## Code availability

The R scripts used for gene expression analysis and GSEA-based visualization were developed using standard packages (including DESeq2 and clusterProfiler) and are available from the corresponding author upon request.
