# Supplementary material for: Sotorasib resistance in KRAS G12C-mutant invasive mucinous adenocarcinoma with implications for VEGF-A
Source: NPJ Precis Oncol. 2025 May 27;9:154. doi: 10.1038/s41698-025-00953-2 (PMC12106728; doi:10.1038/s41698-025-00953-2)
Supplement: Supplementary file 1 — Supplementary_Information [file 41698_2025_953_MOESM1_ESM.pdf]

## Supplementary Information

Supplementary Figure 1

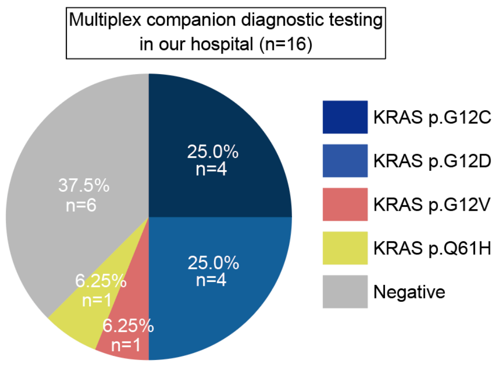


**Supplementary Figure 1. *KRAS* mutation testing results of 16 IMA cases in our hospital.**

Supplementary Figure 2

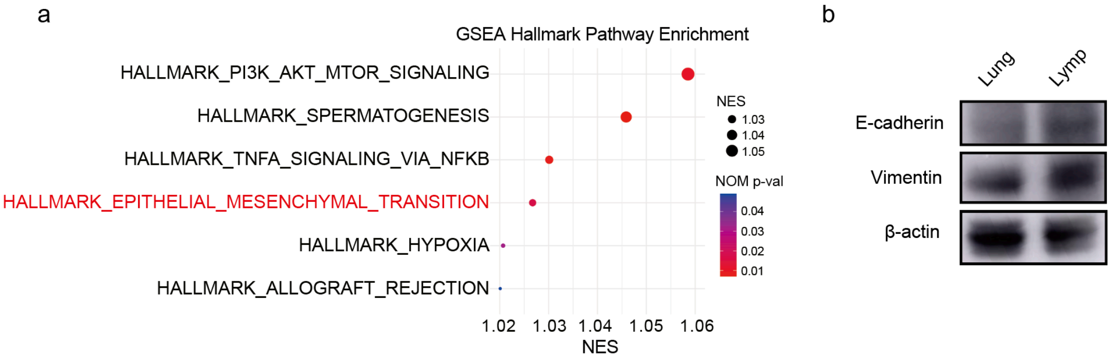


**Supplementary Figure 2. a)** GSEA dot plot for the top 6 gene sets enriched for thoracic lymph node metastasis in autopsy specimens. **b)** Western blot of all lysates extracted from primary lung and lymph node lesions of autopsy specimens.

Supplementary Figure 3

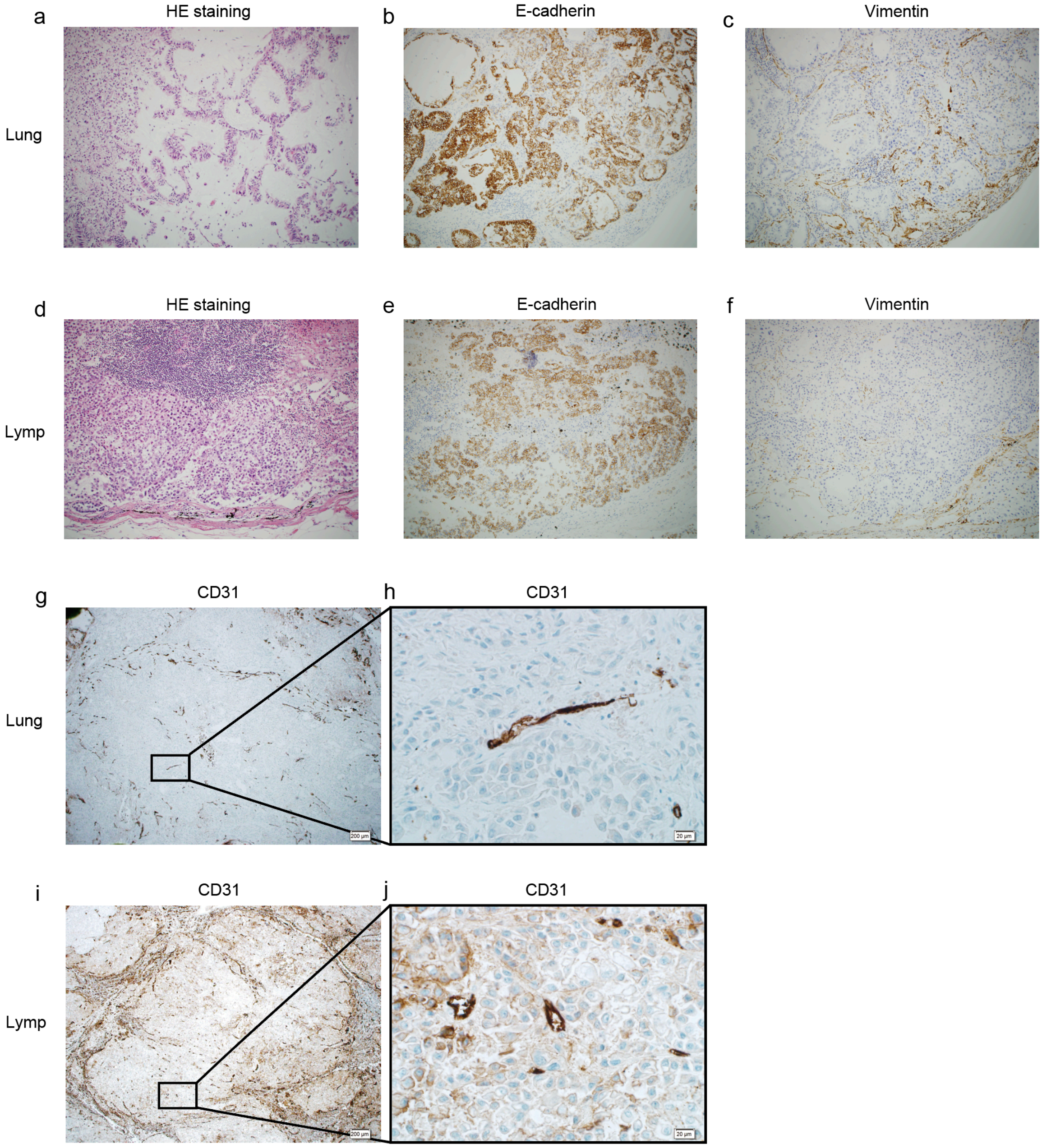


**Supplementary Figure 3. Immunohistochemistry of autopsy specimens. a)** HE staining of primary lung lesions. **b)** E-cadherin staining of primary lung lesions. **c)** Vimentin staining of primary lung lesions. **d)** HE staining of thoracic lymph node metastases. **e)** E-cadherin staining of thoracic lymph node metastasis. **f)** Vimentin staining of thoracic lymph node metastasis. **g)** CD31 staining of primary lung lesions (×4). **h)** CD31 staining of primary lung lesions (×40).

**i)** CD31 staining of thoracic lymph node metastasis (×4). **j)** CD31 staining of thoracic lymph node metastasis (×40).

Supplementary Table 1

| Characteristic (n=32) | |
|---|---|
| **Age** | |
| Median | 72 (56-83) |
| **Gender** | |
| Male | 16 (50.0%) |
| Female | 16 (50.0%) |
| **Smoking history** | |
| Never | 15 (46.8%) |
| Current | 17 (53.2%) |
| **Thoracic lymph node metastasis at diagnosis** | |
| With | 2 (6.3%) |
| Without | 30 (93.7%) |
| **PD-L1** | |
| Negtive | 6 (18.8%) |
| Low | 2 (6.25%) |
| High | 0 (0.0%) |
| Unknown | 24 (75.0%) |
| **Comprehensive genetic profiling** | |
| With | 16 (50.0%) |
| Without | 16 (50.0%) |

**Supplementary Table 1. IMA Clinical data in our hospital.**

Supplementary Table 2

| **Lung** | | | |
|---|---|---|---|
| Allele Name | Gene ID | AA mutaion | Frequency |
| COSM132904 | IL7R | p.T244I | 43.4 |
| COSM5915 | PTEN | Unknown | 45.3 |
| COSM147002 | BLNK | p.P57= | 49.5 |
| COSM516 | KRAS | p.G12C | 38.5 |
| COSM1009 | SMARCB1 | p.S308= | 53.7 |

| **Lymp** | | | |
|---|---|---|---|
| Allele Name | Gene ID | AA mutaion | Frequency |
| COSM132904 | IL7R | p.T244I | 43.4 |
| COSM5915 | PTEN | Unknown | 45.3 |
| COSM147002 | BLNK | p.P57= | 49.5 |
| COSM516 | KRAS | p.G12C | 38.5 |
| COSM1009 | SMARCB1 | p.S308= | 53.7 |
| COSM42924 | SYNE1 | p.E4060D | 49.7 |

**Supplementary Table 2. NGS tissue analysis of the extracted DNA**.
